# Health-related quality of life in long-term survivors of childhood brain tumors: a population-based cohort study

**DOI:** 10.1007/s00520-022-06905-x

**Published:** 2022-03-03

**Authors:** Lisa Ljungman, Tiina Remes, Elisabeth Westin, Alina Huittinen, Tuula Lönnqvist, Kirsti Sirkiä, Heikki Rantala, Marja Ojaniemi, Marika Harila, Päivi Lähteenmäki, Pekka Arikoski, Anna Wikman, Arja Harila-Saari

**Affiliations:** 1 Department of Women’s and Children’s Health, Uppsala University, Uppsala, Sweden; 2 Department of Pediatrics and Adolescence, PEDEGO Research Unit and Medical Research Center, Oulu University Hospital, University of Oulu, Oulu, Finland; 3 Department of Child Neurology, Children’s Hospital, Helsinki University Hospital, University of Helsinki, Helsinki, Finland; 4 Department of Pediatrics and Adolescence, Helsinki University Hospital, Helsinki University, Helsinki, Finland; 5 Department of Neurology, Oulu University Hospital, Oulu, Finland; 6 Department of Pediatrics and Adolescent Medicine, Turku University Hospital, Turku University, Turku, Finland; 7 Pediatric Research Unit, Kuopio University Hospital, University of Eastern Finland, Kuopio, Finland

**Keywords:** Brain tumor, Childhood cancer, CNS tumor, Depression, Health-related quality of life, Radiation therapy, Survivors

## Abstract

**Purpose:**

Survivors of childhood brain tumors (BT) are at high risk for long-term physical and psychological sequelae. Still, knowledge about health-related quality of life (HRQL) and associated factors in this population is sparse. This study investigated HRQL and its predictors in long-term survivors of childhood BT.

**Methods:**

Survivors of childhood BT (mean age = 28.1 years, SD = 6.8, *n* = 60) underwent clinical examination and neurocognitive examination, and completed self-rating questionnaires assessing HRQL (RAND-36) and depressive symptoms (Beck Depression Inventory-II). Socio-demographic information was gathered via a questionnaire. Tumor- and treatment-related information was collected from medical records. Control group data were collected from age-matched controls (*n* = 146) without a history of cancer, randomly selected from the local population registry. Multiple linear regression models were used to investigate predictors of HRQL; separate models were fitted for each domain of the RAND-36.

**Results:**

Male survivors (mean age = 27.0, SD = 6.0, *n* = 39) reported significantly lower HRQL than male controls in the domains of physical functioning, general health, vitality, social functioning, and role limitations-emotional. Female survivors (mean age = 30.2 years, SD = 7.6, *n* = 21) reported comparable levels as female controls in all domains except physical functioning. A higher burden of late effects, not working/studying, being diagnosed with BT during adolescence, and reporting current depressive symptoms were significant predictors of lower HRQL.

**Conclusion:**

Our results highlight that male survivors of childhood BT are at particular risk of impaired HRQL. Also, results point to the close relation between symptoms of depression and impaired HRQL in survivors of childhood BT which should be acknowledged by long-term follow-up care.

## Introduction

The survival rate for childhood brain tumors (BT) in the Nordic countries is approaching 80% [1]. With an increased number of long-term survivors, the focus on late effects and quality of survival has increased accordingly [2]. Compared to survivors of other childhood malignancies, childhood BT survivors are well-known to be at higher risk of serious medical illnesses, functional impairment, and late mortality [3–5]. Medical conditions commonly reported in this population include endocrinological, cardiovascular, and cerebrovascular late effects, as well as cognitive impairment and sensory health conditions [4, 6]. Additionally, BT survivors may suffer from fatigue, pain, emotional disorders, and changes to physical appearance and body image [3, 7]. Compared with healthy controls, BT survivors also report higher rates of attention deficits, peer conflict, social withdrawal, and antisocial behavior [8]. Despite all these well-documented common impairments in physical, neurocognitive, and psychosocial function, firm knowledge about health-related quality of life (HRQL) in long-term survivors of childhood BT is lacking.

HRQL is a multidimensional construct reflecting the impact that health and illnesses may have on well-being with respect to physical as well as emotional, social, and cognitive functioning [9, 10]. The previous literature on the level of HRQL in survivors of childhood BT has been inconclusive, with some studies reporting HRQL in this population to be comparable to healthy controls [11, 12], while others report HRQL to be impaired compared to survivors of other childhood malignancies [13, 14]. It has also been reported that over the course of time, HRQL seems to progressively decline in survivors of childhood BT [15]. Furthermore, previous research has suggested that the domains of social function, physical function, role limitations due to physical problems, general health, and bodily pain seem to be most affected in survivors of childhood BT [16–18].

Regarding predictors of HRQL in long-term survivors of childhood BT, a previous systematic review reported that hypothalamic tumor involvement, osteopenia, need for special education, older age at diagnosis, and radiotherapy (RT) are associated with lower levels of HRQL [19]. Also, children diagnosed and treated during adolescence have been reported to experience a lower HRQL than children diagnosed at an earlier age [20]. Research has shown that having been treated with a ventriculoperitoneal shunt, on the other hand, has been associated with better long-term HRQL [21]. Overall, the effect of treatment variables on HRQL in survivors of BT has, however, been reported to be minor, whereas current health problems have been more strongly associated with decreased HRQL [8]. In particular, current psychosocial, cognitive, and neurological problems have been shown to be related to levels of HRQL [16]. The few previous studies that have investigated HRQL in relation to gender in this population have been contradictive, with some reporting no effect of gender [21, 22] while others have reported that female gender is a predictor of lower HRQL [23]. Since females in general report lower HRQL than males, it is important to use gender-specific control groups and gender-specific norms to interpret the level of HRQL among survivors of BT. Still, in the previous literature in this field, this has not been done, thus hampering conclusions regarding effects on HRQL in this population [24, 25].

Taken together, previous reports on HRQL in long-term survivors of childhood BT have been inconclusive. Inconsistencies in methodology such as the use of non-validated measurements of HRQL and mixed samples with regard to treatment, time of follow-up, gender, and the burden of late effects have been brought up as reasons for these mixed results. The aim of this study was therefore to determine HRQL in male and female long-term childhood BT survivors (> 16 years of age), respectively, by using a well-validated measurement and age- and gender-matched control groups. A further aim was to study the medical, physical, neurocognitive, psychological, and socio-demographic factors associated with HRQL in long-term survivors of childhood BT. By establishing this knowledge, long-term follow-up care can be better adapted to the needs of this population, and individuals at particular risk of low HRQL can be identified and provided with adequate care and support.

## Methods

### Design

The study used a population-based cohort design.

### Participants and procedure

A national cohort of childhood BT survivors who were diagnosed between 1970 and 2008 with BT up to the age of 16 and treated with RT was identified from the registers at Oulu, Kuopio, Turku, Tampere, and Helsinki university hospitals. Additional inclusion criteria for the study were as follows: being at least 16 years of age at the time of the study, having a follow-up time since cessation of all tumor therapies of at least 5 years, and having no other progressive BT known at the time of the study. Eligible participants (*n* = 127) were contacted by letter, including information about the study and its procedure. Potential participants who did not respond were thereafter contacted by phone. The current study is part of a larger study of childhood BT survivors conducted in 2010–2015 (for a more thorough description of the study procedure, please see Remes et al. [26]). Written informed consent was obtained from all participants or their legal guardians.

For the data collection, participants underwent a 2-day clinical examination, including blood samples, craniospinal magnetic resonance imaging (MRI), and neuropsychological examination. Participants also answered survey questions about socio-demographic information and completed self-assessment scales of HRQL, function in daily life, and depression and anxiety. The survey questions and the self-assessment scales were either filled in solely by the participant or, if needed, with some assistance from a caregiver, parent, or a researcher. Medical records were examined to study the treatment of the brain tumor and the possible presence of late effects. Information from medical records was used for classification of potential late effects. For the present study, cross-sectional data from the follow-up assessment were used.

The institutional review boards of the Oulu, Kuopio, Turku, Tampere, and Helsinki university hospitals, Finland, approved the study. The research was conducted according to the principles of the Declaration of Helsinki.

#### Controls

A random sample of age-matched controls was collected from the local population registry. A total of 370 healthy young adults were contacted by letter including information about the study and its procedures. Of these, 146 individuals participated, representing a response rate of 39%. Data from the controls has also been used in a previous study, where they were matched regarding age, gender, and living area to patients included in a previous leukemia study; see Harila et al. [27] for a more thorough description of the procedure for collecting data from the controls. The participants completed the same survey as the survivors including questions about socio-demographic information and self-assessment scales of HRQL, function in daily life, and depression and anxiety.

### Measurements

#### Health-related quality of life

HRQL was assessed using the RAND-36 [24]. RAND-36 consists of 35 items measuring eight dimensions of health: physical functioning (10 items), role limitations caused by physical health problems (4 items), bodily pain (2 items), general health (5 items), vitality (4 items), social function (2 items), role limitations caused by emotional problems (3 items), and mental health (5 items). In the scoring of the questionnaire, item scores are transformed into a 0–100 scale in the 8 dimensions, with higher scores indicating better HRQL. The reliability and the validity of the RAND-36 have been confirmed to be good and the internal consistency of the domains satisfactory, with Cronbach’s alphas > 0.80 for all eight scales [24, 25].

#### Depression

Symptoms of depression were assessed using the Beck Depression Inventory-II (BDI-II) [28]. BDI-II consists of 21 items rated from 0 to 3 total sum ranging from 0 to 63 points with a higher number indicating more depressive symptoms. The items assess symptoms corresponding to criteria for diagnosing depressive disorders according to the *Diagnostic and Statistical Manual of Mental Disorders* (4th ed.; American Psychiatric Association, 1994). The instrument has shown good psychometric properties including high reliability, capacity to discriminate between depressed and non-depressed subjects, and adequate concurrent, content, and structural validity [29].

#### Medical late effects

Potential medical late effects were classified with the Common Terminology Criteria for Adverse Events (CTCAE) version 5.0. With the CTCAE, medical late effects are classified from one to five. One stands for mild or no medical late effects, two for moderate, three for severe, four for immediately life threatening, and five for death caused by a medical late effect. For the present study, the CTCAE was dichotomized when used in the regression models according to “mild or no events” and “moderate or above.”

#### Cognitive function

Cognitive function was assessed using the Performance Intelligence Quotient (PIQ) from the Wechsler Adult Intelligent Scale (WAIS), third version. The scoring of PIQ is based on the normal distribution of the population (mean = 100, SD = 15). Previous research has shown that PIQ is a significant predictor of both educational level and employment status in childhood BT survivors [26].

### Statistical analyses

The level of HRQL was compared between male survivors and male controls in all dimensions of RAND-36 using independent *t* tests. The same procedure was carried out for female survivors and controls, and for comparison of female to male survivors. For survivors, regression models were fitted for each domain of the RAND-36 with the following included as independent variables: gender, age at diagnosis (< age of 13 vs. ≥ age of 13), chemotherapy (yes/no), CTCAE (1 vs. > 1) (yes/no), employment (including being a student) (yes/no), education level (elementary vs. above elementary), relationship status (yes/no), PIQ, and symptoms of depression.

## Results

### Participants

#### BT survivors

Of the 127 potentially eligible participants, 74 individuals participated in the data collection (including clinical assessment, neurocognitive tests, and assessment of psychological function). Of these, 60 (81%) completed the RAND-36 and participated in the present study: 21 (35%) women and 39 (65%) men. There was no difference between responders and non-responders in terms of age at diagnosis, current age, CTCAE, follow-up time, or chemotherapy.

The mean age of the participants was 28.1 years (SD = 6.8). Mean age at diagnosis was 8.5 years (SD = 4.3) and the mean follow-up time was 19.1 years (SD = 5.9). The most common tumor type was the glial cell tumor (45%). All participants had received RT, 63% had been treated with chemotherapy, and 58% had received a ventriculoperitoneal shunt. A minority were married/had cohabiting partnership (27%), 65% (*n* = 39) were working/studying, and 10% (*n* = 6) had a university degree. For the full report of the demographic, clinical, and psychosocial characteristics, see Table 1.

**Table 1:** Demographics and clinical variables for brain tumor (BT) survivors and control group

	BT survivors			Control group		
*n* (%) or mean (SD)	Total (*n* = 60)	Women (*n* = 21)	Men (*n* = 39)	Total (*n* = 146)	Women (*n* = 95)	Men (*n* = 51)
Age	28.1 (6.8)	30.2 (7.6)	27.0 (6.0)	25.0 (5.1)	24.6 (5.4)	25.8 (4.4)
Civil status						
Married	8 (13.3)	6 (28.6)	2 (5.1)	32 (22.1)	20 (21.3)	12 (23.5)
Cohabiting partnership	8 (13.3)	4 (19.0)	4 (10.3)	51 (35.2)	36 (38.3)	15 (29.4)
Not married/cohabiting partnership	44 (73.3)	11 (52.4)	33 (84.6)	62 (42.8)	38 (40.4)	24 (47.1)
Employment						
Working	23 (38.3)	11 (52.4)	12 (30.8)	84 (59.6)	47 (52.2)	37 (72.5)
Studying	16 (26.7)	4 (19.0)	12 (30.8)	34 (24.1)	24 (26.7)	10 (19.6)
Unemployed	10 (16.7)	4 (19.0)	6 (15.4)	10 (7.1)	6 (6.7)	4 (7.8)
Retired	11 (18.3)	2 (9.5)	9 (23.1)	0	0	0
Other	0	0	0	13 (9.2)	13 (14.4)	0
BDI-II^a^	6.8 (7.2)	9.4 (9.8)	5.5 (5.3)	5.7 (9.0)	7.1 (10.0)	2.9 (6.1)
Clinical cutoff for depression^b^	3 (5.2)	2 (10.0)	1 (2.6)	13 (8.0)	10 (10.5)	3 (5.9)
BT survivors variables						
Age at diagnosis	8.5 (4.3)	9.0 (4.6)	8.2 (4.2)			
Follow-up time in years	19.1 (5.9, range 5–33)	20.5 (6.1, range 9–33)	18.3 (5.7, range 5–30)			
Tumor type						
Glial cell tumor	27 (45.0)	13 (61.9)	14 (35.9)			
Embryonal cell tumor	20 (33.3)	6 (28.6)	14 (35.9)			
Other	12 (20.0)	2 (9.5)	10 (25.6)			
Unknown	1 (1.7)	0	1 (2.6)			
Irradiation						
Cranial or cranio-spinal	28 (46.7)	7 (33.3)	21 (53.8)			
Local	32 (53.3)	14 (66.7)	19 (46.2)			
Ventriculo-peritoneal shunt						
Yes	35 (58.3)	11 (52.4)	24 (61.5)			
No	25 (41.7)	10 (47.6)	15 (38.5)			
Chemotherapy						
Yes	38 (63.3)	13 (61.9)	25 (64.1)			
No	22 (36.7)	8 (38.1)	14 (35.9)			
CTCAE^c^						
1	23 (38.3)	5 (23.8)	18 (46.2)			
2	22 (36.7)	7 (33.3)	15 (38.5)			
3	15 (25.0)	9 (42.9)	6 (15.4)			
PIQ	88.4 (13.8)	89.7 (13.9)	87.7 (13.9)			

^a^Norm data on BDI-II in the age group, mean (SD), women = 9.81 (8.67), men = 8.22 (8.06) [30]

^b^Cutoff value of 20 or higher indicative of at least moderate level of depressive symptoms

^c^No scores of 4 or 5 were recorded

*BDI-II*, Beck Depression Inventory-II; *CTCAE*, Common Terminology Criteria for Adverse Events; *PIQ*, Performance Intelligence Quotient

#### Control group

In the control group, 146 individuals participated. Of these, 65% were women (*n* = 95) and 35% men (*n* = 51). The mean age was 25.0 years (SD = 5.1). A majority of the participants in the control group were married or had a cohabiting partnership (57%, *n* = 83), 81% (*n* = 118) were working/studying, and 9% (*n* = 14) had a university degree. See Table 1 for the presentation of the demographic and psychosocial variables for the control group.

### Health-related quality of life

**Fig. 1:**
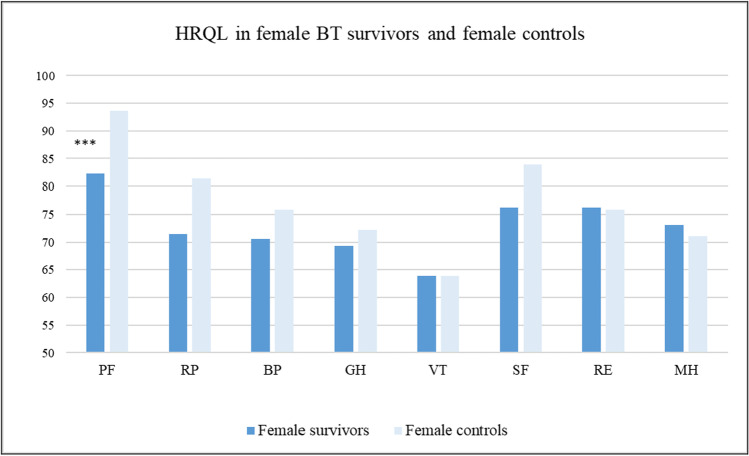
Mean values for the eight RAND-36 domains for female BT survivors and female controls. Significant differences indicated by ***p < 0.001. PF, physical functioning; RP, role limitations caused by physical health problems; BP, bodily pain; GH, general health; VT, vitality; SF, social function; RE, role limitations caused by emotional problems; MH, mental health

**Fig. 2:**
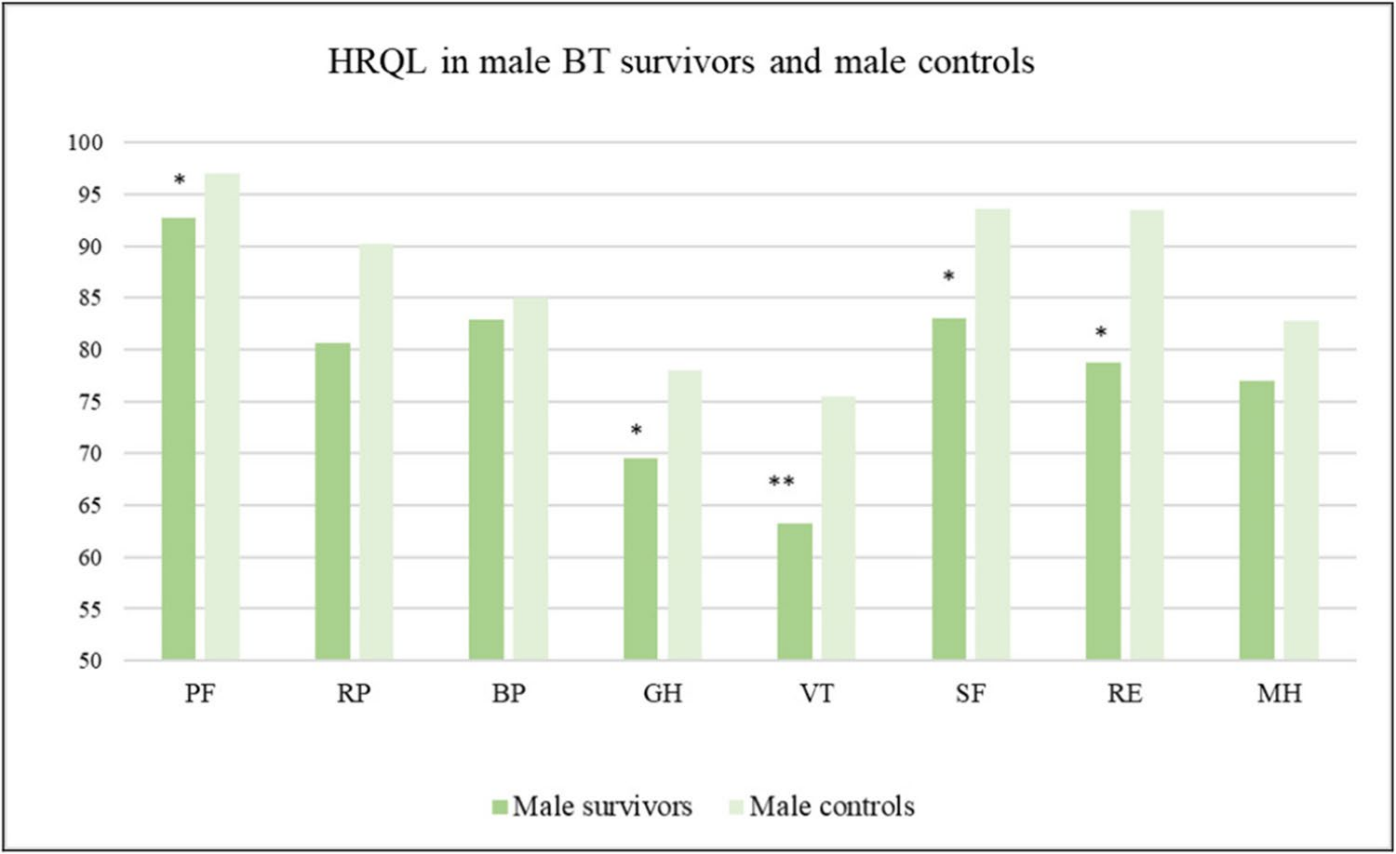
Mean values for the eight RAND-36 domains for male BT survivors and male controls. Significant differences indicated by *p < 0.05; **p < 0.01. PF, physical functioning; RP, role limitations caused by physical health problems; BP, bodily pain; GH, general health; VT, vitality; SF, social function; RE, role limitations caused by emotional problems; MH, mental health

Female survivors’ scores in the dimensions of RAND-36 ranged from 63.9 (vitality) to 82.3 points (physical functioning). Male survivors’ scores ranged from 63.2 (vitality) to 92.7 points (physical functioning). Comparing BT survivors and controls by gender showed that female survivors reported significantly lower HRQL in the dimension of physical functioning (*p* < 0.001) than controls. No other HRQL dimensions differed between female survivors and controls (see Fig. 1). Among men, BT survivors reported significantly lower scores than controls in the following five dimensions: physical functioning (*p* < 0.05); general health (*p* < 0.05); vitality (*p* < 0.01); social functioning (*p* < 0.05); and role limitations caused by emotional problems (*p* < 0.05). No differences were observed in the other dimensions of HRQL for men (see Fig. 2). Lastly, in comparing HRQOL scores between female and male BT survivors, mean values did not differ aside from in the domain physical functioning where males reported significantly higher scores than females (*p* = 0.001). See also Table 2 for a comparison between survivors and controls.

**Table 2:** Descriptive statistics for RAND-36 domains presented by gender. Student’s *t* tests for differences in mean between female BT survivors and female controls, and between male BT survivors and male controls. Bold text indicates a significant *p* value

	Women, mean (SD)			Men, mean (SD)		
RAND-36 domains	BT survivors	Comparison group^a^	*t* test	BT survivors	Comparison group^a^	*t* test
Physical functioning	82.3 (15.0)	93.6 (10.9)	** < 0.001**	92.7 (8.6)	97.0 (7.3)	**0.012**
Role limitations – physical health	71.4 (29.9)	81.4 (28.6)	0.156	80.6 (32.0)	90.2 (26.5)	0.129
Bodily pain	70.6 (26.8)	75.9 (22.8)	0.348	82.9 (20.4)	85.0 (20.6)	0.638
General health	69.3 (17.9)	72.2 (17.4)	0.507	69.5 (19.0)	78.0 (17.5)	**0.029**
Vitality	63.9 (24.1)	63.9 (18.6)	0.990	63.2 (22.9)	75.4 (17.6)	**0.005**
Social functioning	76.2 (18.9)	83.9 (21.7)	0.135	83.0 (22.1)	93.6 (13.3)	**0.010**
Role limitations – emotional problems	71.4 (38.4)	75.9 (33.3)	0.591	78.7 (34.9)	93.5 (22.1)	**0.029**
Mental health	73.1 (21.8)	71.1 (18.4)	0.659	77.0 (16.0)	82.7 (14.1)	0.079

^a^Results for the comparison group were in line with norms for the age group in all domains of the RAND-36 [24]

### Factors associated with health-related quality of life

The multivariable linear regression models showed that receiving the BT diagnosis during adolescence compared to at an earlier age predicted lower HRQL in the dimensions role limitations due to physical problems, social functioning, and role limitations due to emotional problems. Having a score above 1 in CTCAE was associated with lower HRQL in the dimensions general health and vitality. Not working/studying was related to lower HRQL with regard to bodily pain. Lastly, symptoms of depression were related to lower HRQL in all the domains. For full results, see Table 3.

**Table 3:** Multivariable linear regression models for the eight dimensions of RAND-36 reported by unstandardized coefficient B. Bold text indicates a significant *p* value

RAND-36 dimension	Physical function	Role limitations – physical problems	Bodily pain	General health	Vitality	Social function	Role limitations – emotional problems	Mental health
Age at diagnosis	− 0.37 *p* = 0.907	− **26.80** ***p* = 0.003**	− 10.30 *p* = 0.154	− 6.29 *p* = 0.316	− 3.94 *p* = 0.487	− **14.08** ***p* = 0.029**	− **22.93** ***p* = 0.013**	− 2.10 *p* = 0.627
BDI-II	− **0.58** ***p* = 0.004**	− **1.88** ***p* = 0.001**	− **0.93** ***p* = 0.031**	− **1.15** ***p* = 0.003**	− **2.05** ***p* < 0.001**	− **0.98** ***p* = 0.009**	− **3.24** ***p* < 0.001**	− **1.98** ***p* < 0.001**
CTCAE	− 4.76 *p* = 0.101	− 11.39 *p* = 0.146	− 4.75 *p* = 0.448	− **11.41** ***p* = 0.041**	− **13.08** ***p* = 0.011**	5.09 *p* = 0.354	3.38 *p* = 0.671	2.60 *p* = 0.492
Working/studying vs not	− 3.17 *p* = 0.303	9.01 *p* = 0.283	− **15.47** ***p* = 0.025**	1.45 *p* = 0.804	− 1.85 *p* = 0.728	− 6.11 *p* = 0.300	− 3.59 *p* = 0.675	− 3.46 *p* = 0.396

In addition to the variables reported in the table, the following variables were also controlled for in all models: gender, PIQ, education, chemotherapy, partnership, and ventriculoperitoneal shunt. These variables were non-significant for all outcomes

*BDI-II*, Beck Depression Inventory-II; *CTCAE*, Common Terminology Criteria for Adverse Events

## Discussion

In this study, HRQL was investigated in long-term survivors of childhood BT. Results showed that male survivors report significantly lower HRQL than male controls in five of the eight domains of HRQL: physical functioning, general health, vitality, social functioning, and role limitations caused by emotional problems. Female survivors on the other hand reported comparable levels to female controls in all domains, except in physical functioning. Furthermore, our results showed that predictors of low HRQL in long-term survivors of childhood BT include a higher burden of late effects, not working/studying, having been diagnosed with BT during adolescence, and the occurrence of depressive symptoms.

Previous studies have reported either no effect of gender on HRQL [31] or a pronounced negative effect on HRQL among female survivors of childhood BT [23, 32]. In contrast, our findings show that male childhood BT survivors reported more areas of impaired HRQL than female survivors when comparing to gender-matched controls. This difference in findings can be related to methodological issues in previous research such as the use of gender-mixed samples and/or lack of gender-specific comparison groups (e.g., [32, 33]). As females in general report lower HRQL than males [34], which also was the general pattern in our results, the use of these designs may lead to the conclusion that female gender is a risk factor for impaired HRQL following childhood BT. Thus, comparing male survivors to male controls and female survivors to female controls is essential in order to determine the gender effect on HRQL following BT. Importantly, our findings correspond with results from a study using gender-specific comparison data in long-term survivors of childhood cancer (all diagnoses) where depressive symptoms were reported to be comparably higher among male survivors [35].

One factor that should be discussed in relation to the effect of gender on HRQL in this population is the proportion of the male survivors who were in an intimate partner relationship compared to the proportion of female survivors: 15% vs. 48%. These figures correspond with previous results reporting that male BT survivors less often are married compared to female survivors [36, 37]. Also, it should be acknowledged that in the control sample, the difference in prevalence of intimate relationships was smaller (60% among females and 53% among males). As having an intimate partner relationship is a well-known predictor of quality of life and overall well-being [38–40], the lower rate of intimate relationship among male BT survivors could be related to the lower levels of HRQL. Furthermore, and possibly related to this issue, male survivors in our study reported lower levels of HRQL in the area of “social function.” In previous research, it has been shown that survivors of childhood BT assess social aspects to be very important, even more so than functionality, for their quality of life [41]. Thereby, to improve the possibility to develop and maintain relationships, our findings suggest that in particular male survivors could be screened for social ability and function and if necessary offered support interventions such as social skills training.

The results showed that both male and female survivors reported lower scores in the physical functioning domain compared to controls. This finding can be related to the well-known high burden of late effects among survivors [3–5]. In our sample, a large majority of participants were assessed as having at least a moderate burden of late effects, and the results underscore that these late effects have a negative impact on HRQL. Since all participants in the present study had received RT, which is a more intensive treatment than only surgery or chemotherapy, the burden of late effects can be expected to be particularly high in our sample. Still, our findings highlight the need for adequate care and treatment of medical conditions in long-term childhood BT survivors in order to improve HRQL in this population.

In this study, medical, physical, neurocognitive, psychological, and socio-demographic factors associated with HRQL in long-term survivors of childhood BT were examined. Results showed that having a higher burden of late effects, having been diagnosed with BT during adolescence, not currently working/studying, and reporting depressive symptoms were significant predictors of lower HRQL. Our findings replicate previous results showing that older age at diagnosis is a predictor of a lower level of HRQL [19, 20], and thereby highlight that individuals diagnosed with BT during adolescence are at increased risk of long-term impairment of their quality of life. In particular, our results show that BT during adolescence is related to lower HRQL in social function and perceived role limitations due to emotional problems, which should be acknowledged by clinicians. The other treatment-related variables in the analyses (chemotherapy and ventriculoperitoneal shunt) were unrelated to HRQL, which supports the conclusion that treatment variables have a minor effect on the quality of life in long-term survivors [8]. However, previous research has concluded that the treatment with RT is associated to lower HRQL in childhood cancer survivors. In our sample, all participants had received RT. Thereby, RT could not be included as a factor in the analyses. Furthermore, cognitive function, assessed using PIQ, did not predict HRQL. These results contradict previous findings where cognitive function has been associated with lower HRQL in survivors of childhood BT [21]. Our findings thereby suggest that cognitive function per se does not explain the lower HRQL seen in this population, but rather is a factor related to other predictors such as unemployment, which in turn are related to impaired quality of life. Still, it is possible that specific neurocognitive functions would have been related to HRQL; however, it was beyond the scope of this study to investigate further. Lastly, both the burden of late effects and occurrence of depressive symptoms were significant predictors of HRQL. Of particular importance were depressive symptoms, which were associated with all areas of HRQL. This finding is in line with previous results showing that depressive symptoms are strongly associated with impairment in HRQL in other clinical and non-clinical populations. It has even been shown that the effect of depressive symptoms on HRQL is comparable with the effect of the chronic health conditions arthritis, diabetes, and hypertension [42]. Furthermore, it has been suggested that depressive symptoms also may interact with the medical conditions per se to further decrease quality of life, described as an “amplification effect.” Thereby, screening for depressive symptoms should be prioritized in childhood BT follow-up care [43, 44] and by early identification and provision of adequate treatment of depression in this population, improvement in both mental health HRQL, and possibly also medical conditions, could be achieved.

### Strengths and limitations

The current study has several important strengths as well as limitations. First, the control group was not fully age-matched to the survivors. However, as HRQL changes only minimally during young adulthood according to standardized norms of the RAND-36 [30], this difference should be considered insignificant. The control group was not fully matched on the living area of the survivors, which should be acknowledged. Another limitation is the cross-sectional design of the study, hampering conclusions with regard to causality. Future studies using a longitudinal design are encouraged to determine the relationship between HRQL and variables such as symptoms of depression and relationship status. Another possible limitation is the heterogeneous sample in terms of histology of BT and follow-up time in combination with the rather small number of participants, thereby impeding subgroup analyses. Also, since the population of long-term survivors of childhood BT is small, and the sample size in this study therefore also was rather small, the risk of underpowered analyses should be taken into consideration when interpreting the results. Another issue that should be brought to attention is that some of the most functionally and cognitively impaired survivors in the cohort were not able to complete the cognitive testing and/or the BDI-II and the RAND-36, which should be acknowledged in terms of generalizability of the results. Also, as some of the participants with low levels of function were assisted in completing the data collection, this might have induced some bias. Still, we believe this was the preferable choice compared to excluding these participants and missing out on results from them. Lastly, it should be acknowledged that since all participants in the present study had been treated with RT, study results should primarily be generalized to this group of BT survivors. Strengths of the study on the other hand include the very rigorous method for collecting data and the use of validated measurements. Furthermore, the use of gender-specific comparison groups is an important strength of this study. Again, due to the relatively small sample size, however, it was not possible to conduct separate regression analyses for male and female survivors. Future studies should prioritize collecting larger samples and reliable comparison data, for example, by using multinational collaborations, to allow for reliable analyses on the predictors of HRQL in long-term survivors of childhood BT.

## Conclusion

The present study showed that male long-term survivors of childhood BT report significantly lower levels of HRQL than male controls in five of the eight domains of HRQL: physical functioning, general health, vitality, social functioning, and role limitations caused by emotional problems. Female survivors on the other hand report comparable levels to female controls in all domains except in physical functioning. These results should be acknowledged by clinicians and care providers in order to identify survivors who experience impaired HRQL, and to provide adequate support and interventions to allow for improvements in the quality of life among these individuals. Specifically, our results point to the association between low HRQL and a higher burden of late effects, not working/studying, and the occurrence of depressive symptoms, which should be addressed and targeted with appropriate interventions. By providing adequate medical or psychological treatment for symptoms of depression to long-term survivors of childhood BT, HRQL could be improved as well.

## Data Availability

Data are not publicly available due to ethical restrictions. Data that support the findings of this study are available from the corresponding author, upon reasonable request and with necessary ethics approval.
